# Machine learning methods for predicting major types of rheumatic heart diseases in children of Southern Punjab, Pakistan

**DOI:** 10.3389/fcvm.2022.996225

**Published:** 2022-10-12

**Authors:** Sana Shahid, Haris Khurram, Baki Billah, Atif Akbar, Muhammad Ahmed Shehzad, Muhammad Farhan Shabbir

**Affiliations:** ^1^Department of Statistics, Bahauddin Zakariya University, Multan, Pakistan; ^2^Department of Sciences and Humanities, National University of Computer and Emerging Sciences, Chiniot, Pakistan; ^3^School of Public Health and Preventive Medicine, Monash University, Melbourne, VIC, Australia; ^4^Chaudhry Pervaiz Elahi Institute of Cardiology, Multan, Pakistan

**Keywords:** rheumatic heart disease, mitral stenosis, mitral regurgitation, machine learning models, multivariate outliers detection, logistic regression model, deep neural network, random forest

## Abstract

**Objective:**

Rheumatic heart disease (RHD) is a major health problem in the world, particularly in developing countries. This study aimed to predict mitral regurgitation (MR) and mitral stenosis (MS) RHD among children with RHD.

**Methodology:**

Data was collected from the Pediatric Cardiology Department at Chaudhry Pervaiz Elahi Institute of Cardiology Multan, Pakistan from March to October 2019. A sample of 561 children aged 4–14 years, who were diagnosed with RHD of either MR or MS, were recruited from the hospital’s outpatient department. The presence of multivariate outliers was detected, and different machine learning methods, including subset logistic regression, subset logistic regression after deletion, stepwise winsorized logistic regression, robust logistic regression, subset deep neural network, and random forest models were compared using the area under receiver operating characteristics (ROC) curve, sensitivity, and specificity. Parsimony was also considered in model selection.

**Results:**

Out of 561 patients in this study, 75.94% had RHD MR and 24.06% had RHD MS. The average age of study participants was 9.19 ± 2.45 years and of them 55.43% were male. Among the male participants, 58.6 and 45.2% had MR and MS, respectively; and among female participants, those were 70.4 and 29.6%, respectively. Subset logistic regression after deletion appeared as competitive with a discrimination power of 90.1% [95% CI 0.818–0.983]. The sensitivity and specificity of this model were 85.1 and 70.6%.

**Conclusion:**

The best predictive model was subset logistic regression after deletion. The predicted method will be used in the decision-making process, which helps early diagnosis of the disease and leads to prevention. The study findings provide the proper guideline for earlier diagnosis of the RHD MR and MS cases among children with RHD in Pakistan.

## Introduction

Rheumatic heart disease (RHD) is a primary heart problem globally, particularly in developing countries (1–4). RHD is caused by rheumatic fever, an inflammatory disease that can affect many connective tissues, especially in the heart, skin, and brain. It also causes extreme pains in joints, particularly in the knees and ankles. The valves of the heart become sore and can be damaged over time and this can result in leaking and narrowing of the valve of the heart making it harder for the heart to work normally (5). According to WHO, each year an estimated 16 million people suffer from RHD worldwide (6). In developing countries, RHD was a major health burden, causing mortality and morbidity among both children and adults. Among the RHD (lesions), mitral regurgitation (MR) was the most common lesion followed by mitral stenosis (MS) (7, 8). In Latin America, the prevalence of RHD was 1.3/1,000 and in Peru, it ranged from 3.9 to 19.7/1,000 (9). In India, the prevalence of RHD was 1.3–4.5/1,000 (10) and in Bangladesh, it was 1.2/1,000 (11).

In Pakistan, RHD mostly occurred among young adults (12), and the most common lesion was the MR, of which 10.5% had mild, 31.4% had moderate, and 31.4% had severe. MS was the second most common lesion of which 7.0% had mild, 8.1% had moderate, and 55.8% had severe (13). The reported prevalence of RHD in Pakistan was found to be 22/1,000 in school-going children (14). Studies in Bangladesh, India, and Pakistan showed that the common non-clinical predictors of RHD were age, gender, living place, family size, number of people sharing a living room, wall material used in the home, number of siblings, and maternal occupation (4, 15, 16). Further studies conducted in Nigeria showed that the major risk factors of RHD are poverty, malnutrition, overcrowding, poor housing condition, low-socioeconomic status, lack of healthcare facilities, and environmental and genetic factors (2, 17). Several studies identified the factors related to RHD using the simple logistic regression model (3, 8, 15).

None of the previous research identifies the risk factor for the further types of RHD. These factors consist of optimal combinations of clinical and non-clinical symptoms used for the earlier prediction of the disease. These predictions are used in the decision-making process, which helps early diagnosis of the disease leading to prevention.

Furthermore, the presence of multivariate outliers is common in medical data. However, detection of these outliers before the modeling is rare in the literature, especially in generalized linear modeling. Different machine learning models were used in literature to predict heart disease. Prediction of heart disease (18, 19) by machine learning techniques using random forest and artificial neural network. A study (20) also used a random forest model to predict the risk factors of heart disease.

None of the studies focused on the prediction of further types of heart disease in children.

This study aimed to identify the multivariate outliers in data and compare present machine learning algorithms to efficiently predict the RHD MR and RHD MS in children with RHD.

## Materials and methods

### Study design, population, and sample

A cross-sectional and retrospective study design was used to collect data from the outpatient department (OPD) of the Pediatric Department at the Chaudhry Pervaiz Elahi Institute of Cardiology Multan, Pakistan. The data was collected from March to October 2019 and the data was collected from all the accessible children who were present during the given period in the hospital. A sample of 561 children diagnosed with RHD was recruited. This sample achieves more than 80% power.

### Patient’s consent and ethics approval

The study was approved by the Departmental Ethics Committee and boards of Bahauddin Zakariya University, Multan, Pakistan. Written permission was obtained from the hospital. The guardians of each child were informed about the study objectives and data confidentiality and were invited to participate in the study. Upon agreement, they signed a consent form.

### Operational definition of the variables

The data was collected by the principal author. The variables related to demographics, socio-economic status, and general health were collected from children and their parents. While the variables related to patients’ symptoms, clinical measurements, and medical diagnostics were collected from the hospital records.

A brief description of the variables is as follows. The variable gender was classified as male and female, and body mass index (BMI) kg/m^2^ was categorized into underweight (<5th percentile), normal to a healthy weight (5th percentile to <85th percentile), overweight (85th percentile to <95th percentile), and obese (95th percentile or greater) (21). A child is physically active if he/she was playing for at least 60 min a day. A child was considered to have fast food, low-caloric food, and staple food if eating fast food more than once a week, a calorie intake of fewer than 1,200 calories per day, and using cereal grain or tubers as a staple food, respectively.

Access to health care facilities was defined if there was any government hospital or medical unit and government doctor available in their area. Similarly, access to education facilities was defined if there was any government school available in their area. The education level of parents was categorized as uneducated, primary to the middle, middle to higher secondary, higher secondary to graduate, and masters to higher. The occupation of the father was categorized into unemployed or diseased, labor or farmer, private job, business, or civil servant. The dwelling area was categorized into rural and urban. The nutrition was categorized as Good nutrition (protein of more than 5 ounces, fruits up to two cups, vegetables up to three cups, grains up to 6 ounces, and dairy up to 3 cups), normal nutrition (protein up to 5 ounces, fruits up to 1.5 cups, vegetables up to 2.5 cups, grains up to 5 ounces, and dairy up to 2.5 cups), less than normal nutrition is considered poor nutrition. Home environment and parental interaction were categorized into poor, normal, and good. The health condition of other people living in the home was categorized as follows. Good health: no respiratory infections or asthma, no lead poisoning, no injuries, and good mental health. The good quality of health care facilities was defined as well-trained and motivated staff, accurate medical records, water, energy, sanitation, hand hygiene, and waste disposal facilities that are functional, reliable, and safe, and adequate stocks of medicines, supplies, and equipment. The house may consider in good condition if there was good ventilation, sanitation, heating, lighting, facilities for cooking, suitable storage for food, and good access to shops and facilities. The housing tenure was categorized as rented or own house.

The included clinical factors were given here. The heart size was categorized as large and normal; it was written on the children’s file by the doctor after examining the chest X-ray of the child. Whether the child had anemia or not was written on the hospital file by the doctor after examining the complete blood count. The family history of diabetes, smoking status in the family, family history of cardiac disease, sleeping apnea, and wheezing were also written on the children’s file. The echocardiography measurement [which are left ventricular internal-systolic dimension (LVISD) (mm), left ventricular posterior wall dimensions (LVPWD) (mm), left ventricular internal dimension diastole (LVIDD) (mm), left ventricular internal dimension systole (LVIDS) (mm)], and ejection fraction (EF) (%) were retrospectively collected from hospital records. The following blood measures were also collected from hospital records: white blood cell count (WBC) (109/L), red blood cell count (RBC) (1012/L), hemoglobin (HB) (g/dL), hematocrit (HCT) (w), mean corpuscular volume (MCV) (fl), mean corpuscular hemoglobin (MCH) (pg), mean corpuscular hemoglobin concentration (MCHC) (g/dl), platelet counts (109/L), neutrophils (%), lymphocytes (%), monocytes (%), and eosinophils (%).

The outcome variable was RHD type, i.e., RHD MS and RHD MR.

### Data management and analysis

Data were summarized and presented as mean (±standard deviation) for numerical data and relative percentages for categorical data. For statistical modeling and validation, the data were randomly divided into two parts, where the first part (85% of data) was used for model training and the second part (15% of data) was used for testing. The following measurements were used to detect multivariate outliers in the generalized linear model (GLM): Leverage (22, 23), Cook’s Distance (24, 25), Modified Cook Distance (26), Andrew’s Pregibon (27), Covariance Ratio (28), and Welsch’s Distance (29). The cases that were jointly identified by all these methods were considered outliers. For prediction, the following methods were used and compared: stepwise logistic regression (SLR) (30, 31), stepwise winsorized logistic regression (SWLR) where winsorization was detail discussed in literature (32, 33), stepwise logistic regression after deletion (SLRAD), robust logistic regression (RLR) (34, 35) subset deep neural network (sDNN) based on deep neural network (DNN) (36) and random forest (RF) (37). The mathematical and procedural details of the diagnostic measures and models are described in Appendix A in Supplementary material. Finally, using the test data the area under the receiver operating characteristic (ROC) curve, its 95% confidence interval, sensitivity, and specificity were calculated for each model.

R language was used for data analysis. For The sDNN model, we used the *neuralnet* package, and for the random forest model and the *randomForest* package in the R language. All the presented results are based on 10-fold cross-validation.

## Results

The univariate analysis presented in Tables 1, 2 showed that for the RHD MS group, the average age was 8.85 (±2.51) years, 19.6% male and 29.6% female. A total of 71.8% of mothers were uneducated, 25.9% were primary or middle, and 2.2% were secondary or higher educated. A total of 37.0% of fathers were uneducated, 60.0% were primary or middle, and 2.9% were secondary and higher educated. A total of 1.5% of fathers were unemployed, 47.4% were laborers or farmers, 46.7% had small businesses, and 4.44% had private jobs. For the RHD MR group, the average age was 9.30 (±2.44) years, 80.4% male and 70.4% female. A total of 66.4% of mothers were uneducated, 29.1% were primary or middle, and 4.5% were secondary or higher educated. A total of 52.3% of fathers were uneducated, 41.5% were primary or middle, and 6.1% were secondary and higher educated. A total of 62.2% of fathers were laborers or farmers, 36.4% had a small business, 0.47% had a private job, and 0.93% were civil servants. Diabetes in the family and smoking in the family was found higher in the RHD MR group. Meanwhile, family history, wheezing, and the use of staple food were found higher in children having RHD MS. Mostly the fathers of children having RHD MR were labor or former, while the fathers of children having RHD MS had a small business. The children having RHD MR belonged to urban areas, while the children having RHD MS belonged to rural areas.

**TABLE 1:** Descriptive analysis of the categorical variables.

Variable	Category	RHD MR	RHD MS	Variable	Category	RHD MR	RHD MS
Gender	Female	176 (70.4%)	74 (29.6%)	Mother education	Primary/Middle	124 (29.1%)	35 (25.9%)
	Male	250 (80.4%)	61 (19.6%)		Secondary/Higher	19 (4.5%)	3 (2.2%)
BMI	Healthy weight	382 (89.7%)	128 (94.8%)		Uneducated	283 (66.4%)	97 (71.8%)
	Obese	3 (0.70%)	2 (1.5%)	Father education	Primary/Middle	177 (41.5%)	81 (60%)
	Overweight	6 (1.4%)	0		Secondary/Higher	26 (6.1%)	4 (2.9%)
	Underweight	35 (8.2%)	5 (3.7%)		Uneducated	223 (52.3%)	50 (37.0%)
Heart size	Large	219 (51.4%)	68 (50.4%)	Father occupation	Civil servant	4 (0.93%)	0
	Normal	207 (48.5%)	67 (49.6%)		Dead/Unemployed	0	2 (1.5%)
Diabetes	No	247 (57.9%)	102 (75.5%)		Labor/Former	265 (62.2%)	64 (47.4%)
	Yes	179 (42.0%)	33 (24.4%)		Private job	2 (0.47%)	6 (4.44%)
Smoking	No	214 (50.2%)	83 (61.5%)		Small business	155 (36.4%)	63 (46.7%)
	Yes	212 (49.7%)	52 (38.5%)	Dwelling area	Rural	251 (58.9%)	92 (68.1%)
Family history	No	263 (61.7%)	63 (46.7%)		Urban	175 (41.1%)	43 (31.8%)
	Yes	163 (38.2%)	72 (53.3%)	Home environment	Good	96 (22.5%)	13 (9.6%)
Anemia	No	414 (97.1%)	131 (97.0%)		Normal	176 (41.3%)	85 (62.9%)
	Yes	12 (2.8%)	4 (2.9%)		Poor	154 (36.2%)	37 (27.4%)
Apnea	No	235 (55.1%)	92 (68.1%)	Health environment	Good	99 (23.2%)	12 (8.8%)
	Yes	191 (44.8%)	43 (31.8%)		Normal	173 (40.6%)	87 (64.4%)
Wheezing	No	148 (34.7%)	29 (21.5%)		Poor	154 (36.2%)	36 (26.6%)
	Yes	278 (65.3%)	106 (78.5%)	Parental interaction	Good	107 (25.1%)	18 (13.3%)
Inactive	No	328 (76.9%)	112 (82.9%)		Normal	176 (41.3%)	84 (62.2%)
	Yes	98 (23.0%)	23 (17.0%)		Poor	143 (33.6%)	33 (24.4%)
Use of fast food	No	241 (56.6%)	98 (72.5%)	Health care quality	Good	92 (21.6%)	12 (8.8%)
	Yes	185 (43.4%)	37 (27.4%)		Normal	172 (40.4%)	85 (62.9%)
Use of low-calorie food	No	291 (683%)	110 (81.5%)		Poor	162 (38.0%)	38 (28.1%)
	Yes	135 (31.7%)	25 (18.5%)	Health care access	No	163 (38.3%)	41 (30.3%)
Nutrition	Good	102 (23.9%)	13 (9.6%)		Yes	263 (61.7%)	94 (69.6%)
	Normal	184 (43.1%)	85 (62.9%)	Education facilities	No	145 (34.0%)	36 (26.6%)
	Poor	140 (32.8%)	37 (27.4%)		Yes	281 (65.9%)	99 (73.3%)
Staple food	No	194 (45.5%)	55 (40.7%)	Housing tenure	Owned	416 (97.6%)	126 (93.3%)
	Yes	232 (54.5%)	80 (59.3%)		Rented	10 (2.3%)	9 (6.6%)
Income	<10,000	3 (0.70%)	8 (5.9%)	Housing condition	Good	105 (24.6%)	62 (45.9%)
	>20,000	8 (1.8%)	0		Normal	152 (35.6%)	59 (43.7%)
	10000–20000	415 (97.4%)	127 (94.1%)		Poor	169 (39.6%)	14 (10.3%)

**TABLE 2:** Descriptive analysis of the quantitative variables.

Variable	RHD MS	RHD MR	Normal range
Age (Years)	8.85 ± 2.51	9.30 ± 2.44	–
LVISD (mm)	7.50 ± 1.91	6.08 ± 113.00	8–12 mm
LVPWD (mm)	7.13 ± 1.95	6.11 ± 1.51	7–11 mm
LVIDD (mm)	47.53 ± 10.74	45.26 ± 12.53	36–56 mm
LVIDS (mm)	28.45 ± 8.23	27.59 ± 9.44	25–41 mm
EF (%)	63.30 ± 13.32	64.04 ± 11.03	50–70%
WBC (109/L)	8.44 ± 3.01	8.74 ± 3.28	4.0–11.0
RBC (1012/L)	5.20 ± 0.70	5.03 ± 0.79	M (4.5–6.3), F (4.2–5.4)
HB (g/dL)	10.75 ± 1.66	10.67 ± 1.61	M (13.0–18.0), F (11.5–16.5)
HCT (w)	35.69 ± 5.85	34.33 ± 5.73	M (39–52), F (26–40)
MCV (fl)	74.51 ± 6.19	74.66 ± 7.06	77–96
MCH (pg)	25.31 ± 2.56	25.10 ± 3.12	26–32
MCHC (g/dl)	32.60 ± 2.21	32.47 ± 2.27	32–36
Platelet (109/L)	280.49 ± 106.20	295.54 ± 112.01	150–400
Neutrophils (%)	60.72 ± 8.81	59.61 ± 10.19	40–75
Lymphocytes (%)	29.03 ± 8.55	29.60 ± 8.34	20–45
Monocytes (%)	6.13 ± 2.27	5.96 ± 2.47	1–9
Eosinophils (%)	3.89 ± 1.78	3.50 ± 1.73	1–6

**FIGURE 1:**
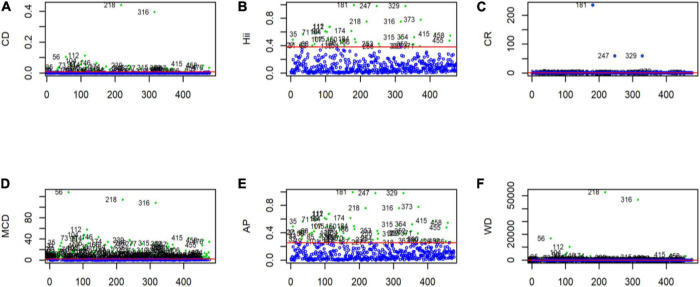
Outliers detected using multivariate outliers detection approach in GLM. **(A)** Outliers detection using the cook distance. **(B)** Outliers detection using leverages. **(C)** Outliers detection using the covariance ratio. **(D)** Outliers detection using the modified cook distance. **(E)** Outliers detection using Andrew’s Pregibon method. **(F)** Outliers detection using Welsch’s Distance. The red marked straight line parallel to the *x*-axis represents the cut-off point. Any observation that falls above this line is considered an outlier.

The result of outlier detection using various outlier detection methods was presented in Figure 1, and according to these methods, there were 13 (2.7%) cases detected as outliers.

**FIGURE 2:**
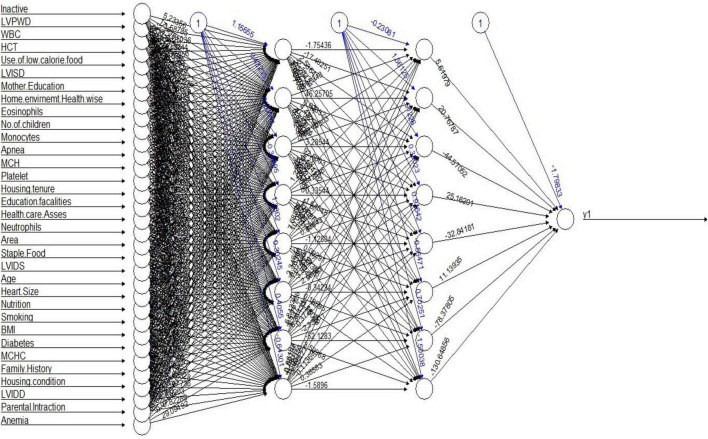
sDNN modeling structure.

The hidden structure and layers of the sDNN model are presented in Figure 2. The sDNN model consists of the first layer, hidden layers, and the last layer. The first layer is the input layer, while the last layer is the output layer, where the explanatory variables are the input variable.

**FIGURE 3:**
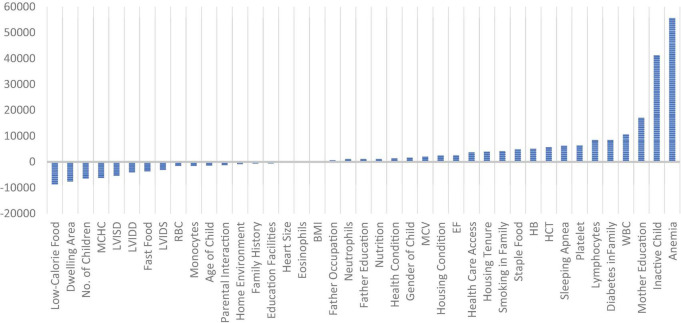
Weights assigned to each variable by the sDNN model.

Figure 3 shows the weights assigned by the sDNN model to each variable, where a higher weight shows the higher importance of the respective variable. The variables anemia had the highest positive weight followed by the physically inactive child, education of the child’s mother, WBC, etc. LVIDS, use of fast food, LVIDD, LVISD, MCHC, number of children in a family, dwelling area, and use of low-calorie food have the highest weights. In the sDNN model, there are 33 input variables, 8 hidden variables, and 1 output variable.

Table 3 shows the results of logistic regression models. The variable that was found significant in all models were LVISD, WBC, male children, wheezing in children, no access to basic health care facilities, and poor and normal housing condition.

**TABLE 3:** Multivariate logistic models using stepwise selection approach.

Variable	Categories^+^	SLR		SLRAD		SWLR		RLR	
		OR	95% CI	OR	95% CI	OR	95% CI	OR	95% CI
Intercept	–	0.003	–	0.001	–	0.000	–	0.050	0.001–2.798
Age	–	1.000	0.9993–1.000	1.000***	0.999–1.000	1.000*	0.999–1.000	1.000***	1.000–1.000
LVISD	–	2.040***	1.6414–2.536	3.121***	2.197–4.432	3.019***	2.237–4.073	3.168***	2.161–4.642
LVPWD	–	–	–	0.751***	0.580–0.974	–	–	0.751***	0.575–0.980
LVIDD	–	–	–	–	–	0.978	0.951–1.006	–	–
WBC	–	1.155***	1.0471–1.274	1.151**	1.033–1.283	1.185***	1.044–1.347	1.097*	0.987–1.220
RBC	–	1.795***	1.1863–2.714	1.818**	1.150–2.875	1.737**	1.132–2.664	–	–
HCT	–	1.069***	1.018–1.123	1.071**	1.015–1.131	1.078***	1.020–1.138	–	–
MCV	–	–	–	1.044**	0.989–1.101	–	–	–	–
Neutrophils	–	–	–	1.026**	0.992–1.062	–	–	–	–
Eosinophils	–	1.224***	1.0397–1.441	1.196**	1.002–1.428	1.276***	1.074–1.516	–	-
Gender	Male	0.515**	0.2862–0.927	0.479**	0.251–0.911	0.540**	0.297–0.979	0.505**	0.268–0.951
Diabetes	Yes	0.480*	0.211–1.094	0.303**	0.117–0.785	0.488*	0.213–1.117	–	–
Smoking	Yes	–	–	0.564**	0.275–1.157	–	–	–	–
Wheezing	Yes	2.445***	1.1049–5.411	3.561**	1.380–9.197	1.996*	0.922–4.316	2.002*	0.900–4.453
Use of fast food	Yes	2.026	0.8454–4.855	2.924**	1.095–7.808	–	–	–	–
Nutrition	Poor	–	–	9.403	0.533–165.808	–	–	–	–
Staple food	Yes	0.242***	0.0889–0.657	0.279**	0.090–0.863	0.287**	0.107–0.771	–	–
Family income	>20000	0.000	–	0.000	–	0.000	–	–	–
	10000–20000	0.027***	0.0009–0.804	0.019**	0.001–0.533	0.025**	0.001–0.503	–	–
Father education	Secondary/Higher	0.432	0.1024–1.819	0.199*	0.038–1.052	–	–	1.517	0.371–6.198
	Uneducated	0.370***	0.1407–0.974	0.307**	0.096–0.980	–	–	0.360*	0.125–1.036
Father occupation	Unemployed	–	–	–	–	2.24*E*+07	–	–	–
	Former	0.217	–	0.019	–	0.460	–	–	–
	Private job	2.983	–	0.351	–	4.059	–	–	–
	Small business	0.238	–	0.013	–	0.526	–	–	–
Health condition	Normal	25.356***	5.8873–109.159	–	–	15.737***	4.400–56.300	–	–
	Poor	7.957*	0.8599–73.662	–	–	3.959	0.512–30.593	–	–
Quality of health care facilities	Normal	–	–	2.948	0.144–60.310	–	–	25.128***	7.210–87.577
	Poor	–	–	0.000	–	–	–	1.835	0.092–36.739
Health care access	No	0.212*	0.0401–1.116	0.085**	0.008–0.884	0.241*	0.047–1.237	0.062**	0.007–0.560
Housing condition	Normal	0.132***	0.0517–0.338	0.197***	0.07–0.550	0.136***	0.052–0.358	0.317**	0.121–0.831
	Poor	0.023***	0.0067–0.079	0.016***	0.004–0.069	0.017***	0.005–0.062	0.011***	0.003–0.051

***Significance at 1%; **Significant at 5%; *Significant at 10%; ^+^Reference categories are: in gender “female”, in diabetes, smoking, wheezing, use of fast food, use of staple food “no”, in nutrition, health condition, quality of health care facilities, housing condition “good”, family income “<10000”, father education “primary/middle”, father occupation “civil servant”, health care access “yes”.

**FIGURE 4:**
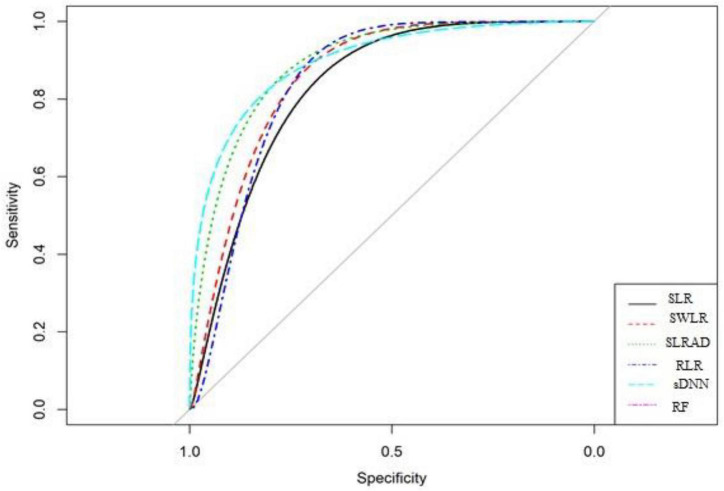
ROC curves for comparison of model performance.

Table 4 and Figure 4 show the comparison of all models. The results showed that the SLRAD model has the highest ROC 0.901 [95% CI 0.818–0.983] with sensitivity and specificity of 85.1 and 70.6%, respectively. The second highest ROC 89.5% [95% CI 0.815–0.974] was for the SWLR model with sensitivity and specificity of 83.6 and 64.7%, respectively. The sDNN model had the third highest ROC 89.2% [95% CI 0.811–0.979] with a sensitivity of 86.5% and specificity of 82.4%. Though there is no significant difference between the ROC of the models (95% CIs are overlapping for all models); however, a tradeoff between ROC and sensitivity/specificity and the number of variables in the model showed that SLRAD is the better model.

**TABLE 4:** Performance comparison of the models on validation data.

Model	ROC	95% CI ROC	Sensitivity	Specificity
SLR	0.877	0.791–0.964	0.851	0.588
SWLR	0.895	0.815–**0**.974	0.836	0.647
SLRAD	0.901	0.818–0.983	0.851	0.706
RLR	0.847	0.716–0.979	0.896	0.706
sDNN	0.892	0.811–0.979	0.865	0.824
RF	0.794	0.673–0.914	1.000	0.588

## Discussion

In this study, the results show that male children are more affected by both RHD MS and RHD MR than female children the previous study also supports our finding (16). The model comparison results show that the best prediction model was SLRAD.

The results of the best-selected model SLRAD show that wheezing contributes 3.56 times, LVISD contributes 3.12 times, RBC contributes 1.81 times, eosinophils contribute 1.19 times, WBC contributes 1.15 times, and HCT contributes 1.07 times in RHD MR as compared to RHD MS. While the risk of RHD MS increases approximately 47% with male children, 30% with diabetes in the family, 27% with staple food, 20% with not a good housing condition [a similar result is also established by the study (1, 3)], and 1.9% with low family income, as compared to RHD MR. The result shows that the poor facilities of health care were a barrier to detecting and diagnosing at an early stage of disease and medication or treatment of RHD, the previous study also supports our findings (12).

Similarly, for the sDNN, the weights of these variables are also significant from our suggested cutoff points. The best selected SLRAD models show some additional variables significant as compared to other models, which are as follows: the use of fast food contributes approximate 2.9 times more is RHD MR as compared to RHD MS. Similarly, the risk of RHD MS increases by approximately 30% when fathers are uneducated and 75% with the risk of LVPWD as compared to RHD MR.

## Conclusion

Improvement in socioeconomic status leads to a significant reduction in RHD MS cases. Therefore, the policymakers/facilitators need to be properly monitored and facilitate those children and families having a low family income, uneducated fathers, no access to basic health care facilities, and poor housing conditions. To control the cases of RHD MR, children should be discouraged for to use fast food. And child suffering from wheezing and non-normal WBC, RBC, HCT, and MCV counts need to be monitored as it has a significant contribution to RHD MR cases. Moreover, the best predictive model is SLRAD. The selected model can help medical practitioners and cardiologists to understand and diagnose the risk factors, their contribution, and risk estimation of RHD MS and RHD MR in children. Thus, the study findings provide the proper guideline to control the RHD MR and MS cases among children with RHD in Pakistan.

## Data availability statement

The raw data supporting the conclusions of this article will be made available by the first and corresponding author, without undue reservation.

## Ethics statement

The studies involving human participants were reviewed and approved by Board of Advanced Studies and Research, Bahauddin Zakariya University, Multan, Pakistan. Written informed consent to participate in this study was provided by the participants or their legal guardian/next of kin.

## Author contributions

SS, HK, BB, and AA contributed to the conception and design of the study. SS and HK organized the dataset, performed the statistical analysis, and wrote the first draft of the manuscript with the help of BB, AA, MAS, and MFS. MAS and MFS reviewed the final draft from a statistical and medical perspective, respectively. All authors contributed to the manuscript revision, read, and approved the final version.
