# Supplementary material for: Machine learning methods for predicting major types of rheumatic heart diseases in children of Southern Punjab, Pakistan
Source: Front Cardiovasc Med. 2022 Oct 12;9:996225. doi: 10.3389/fcvm.2022.996225 (PMC9596762; doi:10.3389/fcvm.2022.996225)
Supplement: Supplementary file 1 [file Data_Sheet_1.docx]

**Appendix A**

**Methods and Models**

**1. Multivariate Outliers Detection Measures for Generalized Linear Model**

Considered the model

$y=f\left(X\right)+\varepsilon$, (1)

As a generalized linear model (GLM), the response is an exponential family member, and *X* is a matrix of order$n\times p$. The observations that impact the regression line's slope are called influential observations and are considered bad outliers. By removing these observations, the estimated coefficients can be significantly changed. The different influence measurement statistics used to detect bad outliers for the GLM are described as follows.

***Leverage*** is the measurement of how far the predictor variable deviates from its mean. The influential observation can be detected with leverage, and cases are declared to be influential if$h_{ii}>2p/n$. Where $h_{ii}$ is the diagonal element of hat matrix *H*. In GLM, the hat matrix is defined as The diagonal elements of $H$is defined as

***Cook's Distance (CD)*** is widely used to detect influential observations in linear regression models. R. Dennis Cook proposed this method in 1977. It measures the complete change in the regression model when the *i*-th observation is removed. The observations $CD\geq4/\left(n-1\right)$are suspected to be influential. For GLM the Cook's Distance is formulated as$CD_{i}=\left(s_{rp_{i}}^{2}\times h_{ii}\right)/\left(p'\times1-h_{ii}\right)$, where$s_{rp_{i}}^{2}$is the Standardized Pearson Residuals and defined as and $r_{p_{i}}$ is the Pearson residual$r_{p_{i}}=\left(y_{i}-\mu_{y}\right)/\sigma_{y}$, were and $\sigma_{y}$ are the mean and standard deviation of the binomial distribution.

***Modified Cook Distance (MCD)*** diagnoses the influential observation more sharply. The observation whose $MCD\geq2\sqrt{\left(n-p\right)/n}$ is suspected to be influential. The MCD for GLM is defined as$MCD_{i}=\left[\frac{n-\left(p+1\right)}{p+1}\frac{h_{ii}}{1-h_{ii}}\right]^{\frac{1}{2}}\left|t_{i}\right|$ where $t_{i}=s_{rp_{i}}\sqrt{\frac{n-\left(p+1\right)}{n-p-s_{rp_{i}}^{2}}}$

***Andrew's Pregibon (AP)*** gives another measure to detect the influential observation, and the observation is declared to be influential observation when$1-AP>2p/n$. The AP measures for GLM are defined as $AP_{i}=1-h_{ii}-\frac{r_{p_{i}}^{2}}{\sum r_{p_{i}}^{2}}$

***The covariance Ratio (CR)*** measures the influence of *i*-th observation on the variances of the estimates. The observation is suspected to be influential when$\left(1+3\frac{p}{n}<CR<1-3\frac{p}{n}\right)$. For GLM the CR is defined as $CVR_{i}=\frac{\left[n-p-s_{rp_{i}}^{2}\right]^{\left(p+1\right)}}{1-h_{ii}}$

***Welsch's Distance (WD)*** is the modified form of DFFITS, and the observation is suspected to be influential if the value of WD is greater than$3\sqrt{p}$. The WD for GLM is defined as $WD_{i}=\left(n-1\right)t_{i}^{2}\frac{h_{ii}}{\left(1-h_{ii}\right)^{2}}$

**2. Stepwise Logistic Regression (SLR)**

Logistic regression is a commonly used model to predict binary outcomes. The logit function of GLM defined in Eq. (1) is as

$\pi_{i}=g^{-1}\left(X\beta\right)$ (2)

Where $X$ is known as $n\times p$ the design matrix and $\beta$ is a $p\times1$ vector of parameters. The binary logistic model for the multiple predictors can be written as

$P\left(y/X_{1},X_{2},...,X_{p}\right)=\frac{e^{\beta_{0}+\beta_{1}X_{1}+\beta_{2}X_{2}+...+\beta_{p}X_{p}}}{1+e^{\beta_{0}+\beta_{1}X_{1}+\beta_{2}X_{2}+...+\beta_{p}X_{p}}}$ (3)

The stepwise logistic regression (SLR) is then evaluated by adopting a backward selection approach. The stopping criteria were chosen on the base of minimum AIC and BIC.

**3. Stepwise Winsorized Logistic Regression (SWLR)**

Winsorization is the method to deal with outliers after truncating the extreme values of the sample data. The process of replacing a specified number of extreme values with a smaller data value is known as Winsorization. To make this model possible for fit, less than$\alpha\%$ and above then $(1-\alpha)\%$ observations are winsorized where $\alpha$is the level of significance for winsorization, which is assumed to be 0.05, so the logistic regression model in Eq. (2) is as

$\pi_{i}=g^{-1}\left(X_{w}\beta\right)$ (4)

where $X_{w}$ is the matrix of winsorized data of order$n\times p$. For applying stepwise winsorized logistic regression (SWLR), a backward selection approach based on AIC and BIC is adopted.

**4. Stepwise Logistic Regression After Deletion (SLRAD)**

The observations commonly diagnosed by all the outlier detection methods are considered outliers. In this approach, the logistic regression model is applied after the group deletion of outliers. Thus, Eq. (1) for logistic regression after deletion is as

$\pi_{i}=g^{-1}\left(X_{\left(n-d\right)}\beta\right)$ (5)

whereas the number of outliers and$X_{\left(n-d\right)}$is the matrix of observation do not contain bad outliers. Finally, the Stepwise Logistic regression after deletion (SLRAD) model was used by following the backward selection approach.

**5. Robust Logistic Regression (RLR)**

Consider the Quasi-likelihood function as

(6)

The solution of this Quasi-likelihood function is the M-estimator of the Mallow type and hence

(7)

while $a\left(\beta\right)=\frac{1}{n}\sum_{i=1}^{n} E\left[v\left(y_{i},\pi_{i}\right)\right]w\left(x_{i}\right)\pi'_{i}$, (8)

and $v\left(y,\pi\right)w(x)=V^{-1}\left(\pi\right)w\left(x,y,\beta\right)\left(y-\pi\right)$ (9)

The Solution of Eq. (6) by using the link function defined in Eq. (1) is the robust M-estimation of GLM. Considered that and where is Huber function and defined as

- (10)

The solution of Eq. (6) by considering Eq. (10) will be the Robust logistic regression (RLR) based on M-estimation using the Huber function. To ensure computational stability, some of the variables were excluded from the robust logistic regression (RLR) model, making the inverse more stable in the estimation process.

**6. Subset Deep Neural Network (sDNN)**

The Artificial Neural Network (ANN) model is considered the machine learning approach and is ideal for predicting disease occurrence in individuals. The model consists of hidden layers that lead the input variables to the output. And if there are many hidden layers in the ANN model it will be generally known as a deep neural network (DNN). The training of the model is done by using backpropagation. There is no need for distributional assumptions of input variables while using this model. Additionally, this model is not much sensitive to outliers in data.

To avoid the over selection of the variables in neural networking we have suggested a method used as a variable selection method. In this procedure, the final weights *w_i_* extracted from the DNN model we will be normalized by their mean. Thus, an *i*-*th* variable will be in the model if$\frac{\left|w_{i}\right|}{\left|\bar{w}\right|}>10\%$. The DNN modeling on a subset using this criterion is said to be a subset deep neural network (sDNN) and it does not affect the results significantly as compared to the fully loaded model.

**7. Random Forest (RF)**

The random forest (RF) model was proposed by Breiman and is also considered the machine learning approach. The random forest can be used for classification and regression. Random Forest is an ensemble of unpruned classification or regression trees created using bootstrap samples of the training data and random feature selection in tree induction. Prediction is made by aggregating the predictions of the ensemble.
